# The Relationships Between Strategies Of Stress Coping And Temperament-Character Traits In Subjects With Bipolar Disorder

**DOI:** 10.1192/j.eurpsy.2022.418

**Published:** 2022-09-01

**Authors:** I. Gundogmus, S. Tekin, A.B. Yasar, Ö. Uzun

**Affiliations:** 1 Kırıkkale Yüksek İhtisas Hospital, Psychiatry, Ankara, Turkey; 2 Gulhane Research and Training Hospital, Psychiatry, Ankara, Turkey; 3 Istanbul Gelisim University, Psychiatry, Istanbul, Turkey

**Keywords:** bipolar disorders, strategies of stress coping, temperament-character traits

## Abstract

**Introduction:**

Bipolar disorder (BD) is a severe mood disorder, which is characterized by a cycling between the mania and major depression. The relationship between coping strategies and temperament-character traits in BD is unclear at this time.

**Objectives:**

The aim of our study was to assess the relationship between strategies of coping stress and temperament-character traits in individuals with BD.

**Methods:**

168 patients diagnosed with BD in full remission were included. All participants were diagnosed by an experienced consultant psychiatrist based on DSM-5 and were assessed with Young Mania Rating Scale (YMRS) for confirmation to remission. Sociodemographic datas of all participants was obtained and Temperament Evaluation of Memphis, Pisa, Paris and San Diego–Autoquestionnaire (TEMPS-A) and Coping with Stress Scale (CSS) were applied.

**Results:**

75 patients (44.6%) were female and the mean age of the sample was 32.64±10.74 years, the mean duration of illness was 8.23±5.52 years and was found that the mean score of YMRS 5.35±4.19. It was presented Table 1 whether there was a statistically significant correlation between TEMPS-A and CSS subscales.

**Conclusions:**

As coping strategies may be related to temperament-character traits and that could be important for psychological interventions in patients with BD.

**Table:** 

	Depressive	Hypertimic	Cyclothymic	Irritable	Anxious
Avoidance	-,067	-,159	,098	-,150	-,083
	,485	,095	,305	,115	,387
Problem-focused coping strategies	**-,268**	-,153	**,366**	**-,246**	-,134
	**,004**	,109	**,000**	**-,009**	,161
Social support	**-,191**	**-,495**	-,060	**-,646**	**-,416**
	**,044**	**,000**	,535	**,000**	**,000**
Total	**-,256**	**-,399**	,149	**-,370**	**-,324**
	**,007**	**,000**	,118	**,000**	**,001**

**Disclosure:**

No significant relationships.

